# Department of Practice of Medicine

**Published:** 1894-07

**Authors:** 

**Affiliations:** Galveston, Professor of Pathology and Lecturer on Mental and Nervous Diseases in the Medical Department of the University of Texas, Galveston


### Current Medical Literature.

### DEPARTMENT OF PRACTICE OF MEDICINE.

EDITED BY PROF. ALLEN J. SMITH, M. D., GALVESTON,
Professor of Pathology and Lecturer on Mental and Nervous Diseases in the Medical Department of the University of Texas, Galveston.

VASOMOTOR ATAXIA.—Dr. S. Solis-Cohen, of Philadelphia (*American Journal of Medical Science*, February, 1894), thus summarizes an article upon the above subject:

1. By the term vasomotor ataxia it is proposed to designate the condition of instability of the mechanism of circulation present in certain persons and characterized by abnormal readiness of disturbance, with tardiness of restoration, of the equilibrium of the cardio-vascular apparatus. The manifestations are most strikingly displayed by the heart and by the peripheral vessels of the extremities, but analogy indicates the occurrence of similar phenomena in the vessels of the glands and viscera, more especially in those of the kidney, of the gastro-intestinal tract, and of the brain. They may occur apparently spontaneously, but often there is a recognizable exciting cause. Among the influences acting as excitants, are temperature, especially cold; toxic agents formed in the body or introduced from without; visceral or reflex excitation, and emotion. The stimulus may be applied centrally or peripherally, but in either case the resulting phenomena indicate a defect of central inhibition, the expression, probably, of functional or nutritional aberration in the great ganglia of the visceral nervous system, in the medullary centers, or in both. The morbid anatomy is uncertain, and the results of necropsies necessarily inconclusive.

2. Vasomotor ataxia may be acquired as a sequela of disease; in many cases it is congenital; in some cases inherited; the condition is not rarely present in several members of a family.

3. In some cases the phenomena are of paretic, in others of spasmodic character. Usually the two kinds of phenomena are displayed in varying degree in the same patient. Whether spasmodic or paretic, the symptoms are suggestive of inco-ordination. They are always in some degree paroxysmal.

4. In exophthalmic goitre, especially such cases as are produced by emotion or are markedly intermittent, is found the extreme type of the "relaxing" variety of vasomotor ataxia.

5. The form of Raynaud's disease known as "local syncope" furnishes an extreme type of the "constrictive" variety, while "local asphyxia" exhibits phenomena of both abnormal relaxation and abnormal constriction of the vessels.

6. Between these extremes are numberless gradations down to the slightest departure from normality, while even the extreme symptom-groups represent merely exaggerations of phenomena that under certain conditions occur in normal individuals.

7. Dermographism is an essential feature of vasomotor ataxia, and in most cases factitious urticaria can be readily produced by cold or by pressure, or by both; mottlings of the skin, certain peculiar markings of the nails, telangiectases, and stigmata, are common.

8. There is usually a hemorrhagic tendency, as shown by ecchymoses, petechiæ, hæmoptysis, hæmatemesis, hæmaturia, and retinal hemorrhage.

9. Even in the absence of hæmaturia, red blood cells are often found in the urine; uric acid, urates and oxalates are likewise common; the presence of albumin, tube-casts and cylindroids is less common, and is usually intermittent. Glycosuria has been observed.

10. In many striking cases there has appeared to be morbid alteration of the thyroid gland.

11. The action of the heart is usually rapid, irregular, and easily disturbed; palpitation is common, and intermittent tachycardia has been noticed. Hæmic and functional murmurs are not uncommon.

12. Among other symptoms and morbid associations observed are anæmia, hysteria, drug idiosyncrasies, urticaria, local œdema, hyperidrosis, angina pectoris, and pseudo-angina, organic heart disease, pulmonary tuberculosis, asthma, hay fever, vertigo, migraine and other forms of headache, transient hemianopia and other visual disturbance, persistent mydriasis, astigmatism, myopia, hyperopia, menstrual irregularities, intermittent polyuria, rheumatism, rheumatoid arthritis, contractures of digits, chorea, epilepsy, neurasthenia, neurotic dyspepsia, gastralgia, enteralgia, and membranous enteritis, most of which are doubtless fundamentally related as effects of a common cause, or as secondary results.

13. In making a diagnosis of simple vasomotor ataxia, it is necessary to exclude primary organic disease. The occurrence of such disease later does not invalidate the original diagnosis. The development of pulmonary tuberculosis in some cases is probably a sequence of vascular and trophic disturbance in the lung. Cardiac hypertrophy and renal lesion may likewise be among the results of disordered circulation.

---

INSANITY AMONG SILK-MILL EMPLOYES.—Dr. W. P. Spratling, formerly assistant physician in the Morris Plains State Insane Asylum of New Jersey (*New York Medical Journal*, May 19, 1894), reports that between January 1, 1887, and December 31, 1893, there were admitted into the Morris Plains Asylum from the silk-mill workers of a single town of New Jersey having less than 100,000 inhabitants, fifty-seven cases of insanity, thirty males and twenty-seven females. Of these, 33 (or 57 per cent.) are attributed to overwork; four have a history pointing to an hereditary origin. The cause is unknown in 13 cases, of which the author is inclined to attribute one half to overwork, thus making a total of 70 per cent. due to physical and mental stress. Nearly 80 per cent. were of acute types, mostly of the form of acute melancholia. Of these cases, at the close of the past year there had been fourteen recorded as cured, five improved, six unimproved, six dead, and twenty-six remaining under treatment as chronic and incurable cases. The unusually large proportion of cases from workers in a single industry, almost constituting an occupation affection, the author attributes to "stress, direct, continuous and powerful," applied in different ways:

long hours spent daily in managing complex and delicate machinery, one person sometimes doing the work of several in order to increase his earnings; insufficient mental relaxation and rest; insufficient outdoor exercise; the accuracy and complexity of manual motion; the mental application constantly required; the vitiated atmosphere and poor food so common to this class of people.

---

INTRODUCTION OF THE EXOTIC DISEASES OF CHILDREN INTO AMERICA.—Dr. J. Lewis Smith, of New York (*American Journal of Medical Science*, March, 1894), calls attention to the fact that our Indians were entirely free from the contagious diseases of childhood, as well as gonorrhœa and syphilis, until the discovery of America by Columbus. It chanced that in the latter part of the fifteenth century, syphilis and gonorrhœa, as yet undifferentiated, first attracted serious attention in Europe under the name of *Morbus Gallicus*, and as the sailors and soldiers of Columbus were affected by it, an absurd theory for the time prevailed that the disease had been communicated to them by the natives of the West Indies—the opposite being in reality the truth.

The low class of adventurers who surrounded and succeeded Columbus introduced small-pox into the island of Hispaniola eleven years after the death of Columbus (1506). Cortez, however, carried with him from Cuba the negro who was his most powerful weapon in the conquest of Mexico; from this one negro arose the appalling epidemic of variola that rendered the Mexicans such easy victims to the conqueror. Dr. Moore is quoted as stating that 3,500,000 natives were destroyed by the virulence of the disease in this one kingdom. Later, through the agency of William Penn's colonists, the disease was introduced into Pennsylvania (1682), the malady having broken out in one of Penn's vessels, the Welcome, during the passage.

Scarlet fever and measles were not differentiated one from the other until two centuries after the discovery of America; hence the term "measles," applied to both, may have meant either one in referring to an epidemic disease which appeared in the West Indies soon after their occupation by the Europeans. However, the author concludes from the evidence examined that scarlatina did not appear in the western hemisphere until within the last two centuries. In 1635, in Kingston, a town about forty miles east from Boston, occurred the first case of diphtheria, and coincident with the first epidemic of this disease, which followed, was also one of scarlet fever. The latter again disappeared, or became so rare that it was not again mentioned in medical literature in this country until a century later, when the first epidemic of scarlet fever in Philadelphia (1774) occurred. Subsequently it became frequent in America. Professor Louis Thomas, in his monograph in Ziemssen's Encyclopedia, states that this disease spread from Europe over America.

Measles was described by the Arabic school of medicine as early as the tenth century. An epidemic of this disease occurred among the Indians in the first decade following the discovery of the continent.

In the history of the Kingston epidemic of diphtheria, in 1635, it is interesting to note that the first forty cases attacked by it perished. This dread disease extended westward, but it took years before it reached the Hudson, probably because of the slowness and difficulties of travel. A widespread epidemic of some throat distemper was described on Long Island just before the Revolution, but no epidemic of diphtheria is mentioned from the time of the Revolution until the end of the century; in fact, it was not until the decade following 1850 that it made its appearance to any extent; since then it has become established and endemic in our towns.

